# Exploration of the Tolerance of Novel Coronaviruses to Temperature Changes Based on SERS Technology

**DOI:** 10.3390/bios15090558

**Published:** 2025-08-22

**Authors:** Yusi Peng, Shuai Zhao, Masaki Tanemura, Yong Yang, Ming Liu

**Affiliations:** 1 State Key Laboratory of High-Performance Ceramics and Superfine Microstructures, Shanghai Institute of Ceramics, Chinese Academy of Sciences, 1295 Dingxi Road, Shanghai 200050, China; pengyusi@mail.sic.ac.cn (Y.P.); zhaoshuai211@mails.ucas.ac.cn (S.Z.); 2 Center of Materials Science and Optoelectronics Engineering, University of Chinese Academy of Sciences, Beijing 100049, China; 3 University of Chinese Academy of Sciences, No.19 (A) Yuquan Road, Beijing 100049, China; 4 Department of Frontier Materials, Graduate School of Engineering, Nagoya Institute of Technology, Showa, Nagoya 466-8555, Japan; tanemura.masaki@nitech.ac.jp; 5 Department of Orthopedics, Shanghai Fourth People’s Hospital, School of Medicine, Tongji University, Shanghai 200333, China

**Keywords:** SERS technology, Au nanoarrays, SARS-CoV-2 virus, temperature tolerance

## Abstract

Motivated by the rapid development of SERS technology, trace detection of various viruses in the sewage and body fluid environments and accurate positive and negative diagnosis of detection samples can be achieved. However, evaluating the environmental survival ability of viruses based on SERS technology remains an unexplored issue, but holds significant guiding significance for effective epidemic prevention and control as well as inactivation treatment. In this work, Au nanoarrays were fabricated on silicon substrates through a simple Ar ion sputtering route as ultra-sensitive SERS chips. With the synergistic contribution of the “lightning rod” effect and the enhanced coupling surface plasmon caused by the nanoarrays, the ultra-sensitive detection of SARS-CoV-2 S protein with a concentration of 1 pg/mL and SERS enhancement factor of 4.89 × 10^9^ can be achieved. Exploration of the environmental survival ability of the SARS-CoV-2 virus indicates that the Raman activity of SARS-CoV-2 S protein exhibited higher temperature tolerance from 0 °C to 60 °C than SARS-CoV S protein, suggesting that the SARS-CoV-2 virus has less temperature influence from increasing air temperature than the SARS-CoV virus to a certain extent, which explains the seasonal recurrence pattern and regional transmission pattern of the novel coronavirus that are different from the SARS virus.

## 1. Introduction

Since the SARS-CoV virus that broke out in 2002, to HCoV-HKU1 in 2005, MERS-CoV in 2012, and then to the SARS-CoV-2 virus that rapidly broke out and swept the world in 2019, respiratory diseases caused by coronaviruses have been seriously threatening human life and health. Especially the highly contagious and lethal SARS-CoV-2 virus that caused millions of deaths has attracted great attention to the pathological research and detection technology of viruses [1,2]. Although both the SARS-CoV virus and the SARS-CoV-2 virus belong to coronaviruses, the former disappeared after about nine months, while SARS-CoV-2-infected cases still exist to this day. The core difference lies in the significant differences in their transmission characteristics, pathogenic mechanisms, and environmental adaptability. Among them, the research on the environmental adaptability of highly contagious viruses is of great significance for taking effective epidemic prevention and control measures [3].

Variants of SARS-CoV-2 viruses have been confirmed to have a stronger immune escape ability, enabling them to repeatedly infect the population. Additionally, the SARS-CoV-2 virus also demonstrates a stronger survival ability in the natural environment. For instance, the SARS-CoV virus dies after 8 h on cardboard, while the SARS-CoV-2 virus can still be detected alive after 72 h. More importantly, the survival ability of the SARS-CoV virus significantly declines at high temperatures, and its infectivity is notably reduced at 38 °C and 95% humidity, which might be one of the reasons why the SARS-CoV virus disappeared in the summer of 2003, while the SARS-CoV-2 virus can survive for 5 to 10 days at room temperature of 24 °C. Research has shown that the SARS-CoV-2 virus has relatively high stability in a liquid environment at 4 °C (such as virus transport culture medium) with no significant decrease in infectivity within 14 days, and can be stored for several years at −70 °C [4]. The SARS-CoV-2 virus can survive for more than three weeks in environments below 0 °C such as cold chains [5,6]. Furthermore, research on the tolerance of the SARS-CoV-2 virus to high temperatures indicated that with rising temperatures, the resistance of virus to temperature decreases. The SARS-CoV-2 virus can be inactivated when heated at 65 °C for 90 min or at 75 °C for 30 min. When SARS-CoV-2 viruses are kept at 37 °C for one day, their activity drops to 0.1%. When SARS-CoV-2 viruses are kept at 37 °C for two days, these viruses can be completely inactivated, which corresponds to the phenomenon that the seasonal regression of the SARS-CoV-2 virus is not obvious. Moreover, some variant strains of the SARS-CoV-2 virus, including Delta and Omicron, can be completely inactivated when kept at 56 °C for 30 min, 70 °C for 5 min, and 95 °C for 10 min [4]. Furthermore, the research group of Gao Feng revealed the differences in the binding characteristics of the SARS-CoV-2 virus and SARS-CoV virus to the human receptor ACE2 at different temperatures through molecular dynamics simulations [7]. It was found that the RBD structure of the SARS-CoV-2 virus is more stable than that of the SARS-CoV virus, and the SARS-CoV-2 virus has a stronger binding ability to the ACE2 receptor. Moreover, the binding ability of the SARS-CoV-2 virus to ACE2 did not significantly decrease in a temperature environment of 50 °C, which might be originated from the fact that the SARS-CoV-2 virus has stronger environmental adaptability. The above research conclusions provide a solid theoretical guidance for the prevention and control of the SARS-CoV-2 virus epidemic and inactivation treatment.

In current experimental research, the in vitro detection of viruses is mainly achieved through nucleic acid detection using fluorescence quantitative qPCR technology [8]. The biochemical stability of viruses in vitro is explored through virus culture. The three-dimensional structural changes of viral proteins under temperature changes are analyzed by the cryo-electron microscopy and X-ray crystallography techniques to evaluate thermal stability of viruses. However, these techniques usually require professional technicians, complex and time-consuming experimental procedures, professional and expensive experimental equipment, and strict experimental conditions. Therefore, it is necessary to develop faster and simpler research methods to obtain the environmental adaptability characteristics of sudden, highly contagious, and highly pathogenic unknown viruses in time. Surface-enhanced Raman scattering (SERS) technology, as a trace spectral detection technology, has been widely applied in environmental analysis [9,10], food safety [11,12], biosensing [13,14,15], and other fields [16], especially in the detection of infectious pathogens due to its advantages of high sensitivity, fast response, and spectral fingerprint characteristics [17,18]. Research on diagnosing cervical cancer by unveiling the biochemical fingerprint of cancerous cervical cells based on label-free SERS detection technology has demonstrated the application potential of SERS detection technology in the field of biosensing [19]. The research groups of Yang and Du were the first to apply SERS technology to obtain the standard Raman fingerprint of the SARS-CoV-2 S protein and accurately identify the Raman peaks, as well as achieve the diagnosis of SARS-CoV-2 virus particle integrity and infectivity [20,21,22,23,24]. In 2020, Y.T. Yeh et al. reported a microfluidic platform for rapid label-free capture and detection of viruses, which integrated Au nanoparticles and carbon nanotube arrays as filter pores for SERS detection and virus capture. This label-free SERS platform can obtain the Raman signals of 100 to 1000 virus particles, demonstrating a rather high sensitivity [25]. J. Choo et al. reported an aptame-modified SERS sensor that can complete the detection of the SARS-CoV-2 virus within 15 min with a limit of detection (LOD) better than 10 PFU/mL [26]. Another research team from Nanyang Technological University in Singapore has designed a breath analysis module based on SERS technology that can screen for SARS-CoV-2 viruses within five minutes with a detection sensitivity better than 95% [27]. D. Paria et al. developed SERS sensors for SARS-CoV-2 viruses using large-area nanoimprint lithography technology [28]. The group of Xiao has developed a lateral flow immunoassay method combined with SERS technology to simultaneously detect SARS-CoV-2 IgM and IgG, and the detection sensitivity is 800 times higher than that of the simple LFIA technology [29]. In conclusion, trace detection of various viruses in the sewage and body fluid environments and accurate positive and negative diagnosis of detection samples can be achieved based on SERS technology. However, evaluating the environmental survival ability of viruses based on SERS technology remains an unexplored issue at present.

In this work, the SARS-CoV-2 virus and SARS-CoV viruses, which have a biological homology as high as 74.6%, are adopted as research objects to explore their tolerance to temperature changes. Firstly, a structure of Au nanoarrays that can significantly enhance the Raman signal of the target virus was constructed as ultra-sensitive SERS chips based on magnetron sputtering technology. Based on the developed Au nanoarray SERS chips, Raman spectra of SARS-CoV and SARS-CoV-2 S protein, SARS-CoV-2 S pseudovirus, and N pseudovirus under temperatures ranging from 0 °C to 200 °C were measured to check their temperature tolerance. Furthermore, the SARS-CoV and SARS-CoV-2 S proteins on the SERS chips were subjected to heating and then cooling treatment to explore the tolerance of the protein structure to temperature changes.

## 2. Experimental Methods

### 2.1. Preparation of Au Nanoarray SERS Chips

Referring to the construction route of noble metal nanoarray structures reported in our previous works [22,30], Au nanoarray SERS chips were fabricated by Ar ion beam sputtering technology. Firstly, the silicon wafers with crystal orientation of (001) and resistivity of 1^−10^ Ω were ultrasonically treated in acetone for 10 min, then washed with deionized water and dried under a nitrogen flow. Subsequently, the silicon wafers were transferred to piranha solution and immersed for 1 h, then washed with deionized water and dried under a nitrogen flow again. Based on a radio-frequency helical magnetron sputtering system (ULVAC Co., Ltd., Shanghai, China, MPS-2000-HC3), Au films with different thicknesses were prepared by sputtering the high-purity Au targets (99.99%) into high-purity Ar gas. The thickness of Au films was controlled by the sputtering time. The required amount of pure Ar (99.9995%) gas was introduced through the mass flow controller to achieve the required deposition pressure. Au nanoarray structures were fabricated by an ion beam system equipped with a Kaufman-type Ar ion gun with 1 keV (Iontech.Inc. Ltd., Beijing, China, model MPS 3000 FC). Before sputtering, a thin carbon film was deposited on the Au film to promote the formation of ion-induced nanoarray structures. Ar ion sputtering was conducted at room temperature for 4 to 10 min with the base pressure and working pressure of 5.0 × 10^−5^ and 2 × 10^−2^ Pa, respectively. Finally, the residue of the carbon layer was removed by ethanol ultrasonic treatment to obtain the pure Au nanoarray structures.

### 2.2. SERS Performance Characterization of Au Nanoarrays

To explore the SERS performance of Au nanoarrays, SARS-CoV-2 S protein samples with concentrations of 10 ng/mL, 1 ng/mL, 100 pg/mL, 10 pg/mL, and 1 pg/mL were configured for Raman detection. Meanwhile, the Raman spectra obtained from the direct Raman detection of SARS-CoV-2 S protein powder were used as the standard control spectra. An amount of 20 μL of solution containing SARS-CoV-2 S protein was dropped onto the SERS chips, and then placed in a vacuum-drying chamber at 45 °C for drying. All Raman spectra were detected by the mapping scanning technology in a Renishaw inVia Reflex Raman spectrometer. The laser beam was focused by a 50× microscope objective lens, the laser power was 300 mW × 100%, the excitation wavelength of laser was selected to be 785 nm, the cumulative time was set to 1 s, and the Raman Mapping scanning area was 0.2 mm × 0.1 mm with a step size of 10 μm to obtain approximately 200 test points.

### 2.3. Temperature Influence on the SARS-CoV and SARS-CoV-2 S Proteins, SARS-CoV-2 S and N Pseudovirus

SARS-CoV-2 S protein and SARS-CoV S protein with a concentration of 100 ng/mL were dipped on the surface of Au nanoarray SERS chips, respectively. These samples were treated in incubators with different temperatures ranging from −20 °C to 200 °C and measured in situ by a Ranishaw Invio Raman Spectrometer. During the Raman-temperature recovery tests, the temperature was continuously increased from 0 °C to 50 °C, 60 °C, 70 °C, 100 °C, and 200 °C, and kept for 10 min at every stage. Then, the temperature was recovered to 40 °C, 50 °C, 60 °C and 50 °C, 50 °C, 50 °C, respectively. All Raman tests were conducted at every temperature point and on the same substrates.

## 3. Results and Discussion

### 3.1. Development of Human ACE2-Modified SERS Chips

**Figure 1:**
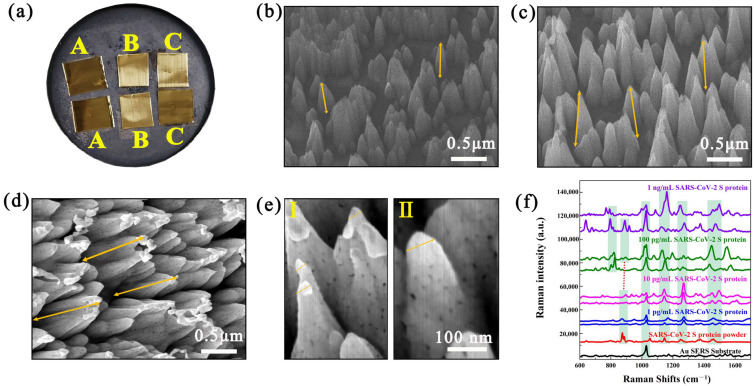
(**a**) Actual pictures of Au nanoarray SERS chips with Ar ion sputtering for 100 s (A), 300 s (B), and 400 s (C). (**b**–**d**) SEM images of Au nanoarray SERS chips with Ar ions sputtering for 100 s (**b**), 300 s (**c**), and 400 s (**d**). And the yellow arrows are used to measure the length of nanoarrays. (**e**) High-resolution SEM image of the vertex of Au nanoarray SERS chips. Ⅰ and Ⅱ in (**e**) represent the smaller and larger tip morphologies of nanoarrays, respectively. (**f**) Raman spectra of 1 ng/mL~1 pg/mL SARS-CoV-2 S protein on Au nanoarray SERS chips.

In this work, a novel nanoarray structure was designed and constructed to significantly enhance the Raman signal of antigens through multi-level SERS enhancement effects. Based on previous work [8], the designed high-density nanoarrays with appropriate structural parameters can form nanoforest structures similar to “virus traps”, capable of capturing viruses with sizes ranging from 50 to 200 nm. This nanoforest structure can induce viruses to easily fall into the nanoarrays and prevent them from escaping. The tips of nanoarrays can generate a “lightning rod” effect, and the Au nanoarrays can induce multiple scattering of incident laser light to stimulate an enhanced electromagnetic coupling effect. Firstly, two-dimensional Au nanoarray structures with different lengths were fabricated by regulating the Ar ion sputtering time based on magnetron sputtering technology. The actual picture and SEM images are shown in Figure 1a–d. It can be clearly observed that as the Ar ion sputtering time increases, the length of nanoarrays also increases. When the Au film was sputtered by Ar ion for 100 s, 300 s, and 400 s, the lengths of the nanoarrays were approximately 450 nm, 700 nm, and 950 nm, respectively. When the Au film was sputtered by Ar ion for 400 s, the diameter of the nanoarray stem was approximately 400 nm, and the interval between the nanoarrays was about 50–1000 nm. The high-resolution SEM image in Figure 1e shows the vertex morphologies of several nanoarrays. The tip of the nanoarray exhibits an extremely sharp curvature with a vertex diameter of approximately 30 to 80 nm, which is conducive to generating a “lightning rod” effect to significantly enhance the Raman signal of the target molecules being detected. Based on the morphology characteristics of Au nanoarrays with strong local surface plasmon resonance (LSPR), even if the R6G molecules were diluted to a low molar concentration of 10^−10^ M, an obvious Raman signal of R6G molecules could still be detected by adsorbing molecules to Au nanoarray SERS chips (Figure S1). Moreover, it was found that the linear relationship of Raman intensity at 1647 cm^−1^ was satisfactory in the range of 10^−5^ M to 10^−10^ M with a correlation coefficient of 0.992, demonstrating the excellent SERS performance of the developed Au nanoarray SERS chips.

In order to explore the SERS performance of Au nanoarrays, SARS-CoV-2 S protein solutions with concentrations of 1 ng/mL, 100 pg/mL, 10 pg/mL, and 1 pg/mL were configured as detection samples. The SERS chips adsorbed with SARS-CoV-2 S protein were scanned and Raman detected by mapping scanning technology. Because the diameter of the SARS-CoV-2 virus is approximately 100 nm which is much larger than the size of conventional detection molecules, the surface S protein of the SARS-CoV-2 virus will occupy the SERS-enhanced region on the surface of Au nanoarrays, thereby obtaining the detected Raman spectra of the SARS-CoV-2 S protein [31]. As shown in Figure 1f, the SERS-enhanced Raman peaks of SARS-CoV-2 S protein with concentrations ranging from 1 ng/mL to 10 pg/mL can be matched well with the Raman peaks of SARS-CoV-2 S protein powder. When the concentration of SARS-CoV-2 S protein was as low as 1 pg/mL, the characteristic Raman peaks belonging to the SARS-CoV-2 S protein still appeared in the Raman mapping, indicating that the LOD of Au nanoarray SERS chips is as low as 1 pg/mL. Taking the Raman peak of SARS-CoV-2 S protein with concentration of 100 pg/mL at the Raman shift of 1048 cm^−1^ as the standard, the calculated EF is approximately 4.89 × 10^9^.

### 3.2. The Temperature Tolerance of Raman Activity for SARS-CoV S and SARS-CoV-2 S Protein

Theoretically, when the temperature rises from room temperature to 80 °C and then to 140 °C, the surface enhancement Raman effect becomes more and more obvious. This is because an increase in temperature will lead to an enhancement of molecular vibration and an increase in the number of excited states, as well as an increase in the cross-sectional area of the transition from a higher vibrational energy level, resulting in an increase in Raman scattering intensity as the temperature rises. Meanwhile, an increase in temperature will enhance the molecular collision rate, thereby causing the Raman peaks to widen [32,33]. The research group of Di Fabrizio investigated the structural changes of different proteins, lysozyme, Ribonuclease-B, bovine serum albumin, and myoglobin, in the temperature range between 65 and 90 °C. Even for small temperature differences conformational changes can be observed, which can be attributed to the effect of thermodynamic perturbation to the Raman spectra of proteins [34].

**Figure 2:**
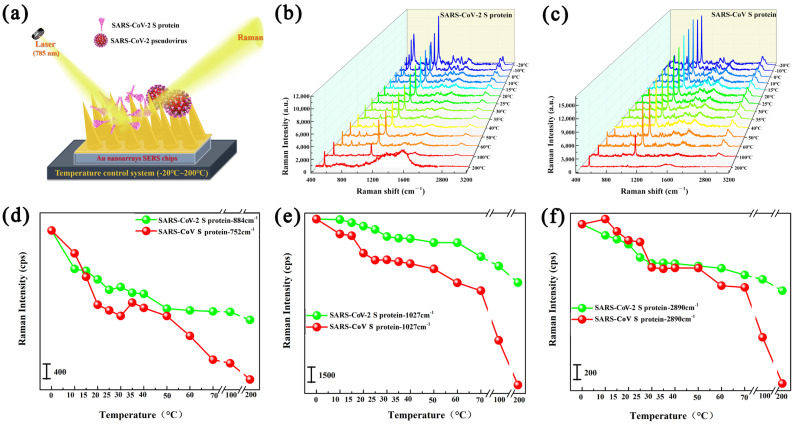
(**a**) Raman scattering schematic diagram of SARS-CoV-2 S protein and SARS-CoV-2 pseudovirus enhanced by Au nanoarray SERS chips. (**b**,**c**) Raman spectra of (**b**) SARS-CoV S, (**c**) SARS-CoV-2 S proteins with concentration of 100 ng/mL measured under temperatures ranging from 0 °C to 200 °C. (**d**–**f**) The Raman intensity changes of SARS-CoV S and SARS-CoV-2 S protein with the temperature ranging from 0 °C to 200 °C at the Raman shifts of (**d**) 884 cm^−1^/752 cm^−1^, (**e**) 1027 cm^−1^, (**f**) 2890 cm^−1^.

The above literature indicates that Raman spectroscopy has the ability to detect the advanced structure of proteins. Therefore, we examined the Raman spectra of SARS-CoV and SARS-CoV-2 spike protein under temperatures ranging from 0 °C to 200 °C based on the developed Au nanoarray SERS chips, to check their temperature tolerance. The Raman scattering schematic diagram is shown in Figure 2a. The Raman spectra of SARS-CoV and SARS-CoV-2 S protein were measured under temperatures ranging from 0 °C to 200 °C, as shown in Figure 2b,c. The SARS-CoV-2 and SARS-CoV S proteins exhibited some distinctly characterized Raman bands at 752 cm^−1^, 884 cm^−1^, 1027 cm^−1^, and 2980 cm^−1^ originating from the vibration modes of aromatic ring in Tryptophan (Trp), the N-H and C-H deformation modes of aromatic ring in Tryptophan (Trp), the N-H in-plane deformation in Phenylalanine (Phe), the C-H stretching modes in CH, CH_2_, and CH_3_ of the aliphatic group, and C-H and N-H stretching modes in the skeleton, respectively [35,36,37]. Therefore, the Raman intensity of these four distinctly characterized Raman peaks was selected to evaluate the temperature tolerance of SARS-CoV-2 and SARS-CoV S proteins. Interestingly, the intensity of the mainly distinct Raman bands for two kinds of S protein both exhibited a slow downward trend from 0 °C to 60 °C, and a sharp downward trend from 60 °C to 200 °C, as shown in Figure 2d–f and Table S1. The intensity of several characterized Raman bands for SARS-CoV S exhibited a gently downward trend in intensity from 0 °C to 50 °C, and decreased very sharply from 50 °C to 200 °C. Normally, the intensity of Raman bands for protein crystal will decrease slightly with increased temperature in the range of 20~60 °C. Removing this elementary effect of temperature on crystal Raman intensity, the variation trend of SARS-CoV S Raman intensity represented the vibrational strength and molecular activity, exhibiting almost the same change for which the SARS outbreak became weaker with the increase in air temperature in 2003. However, the intensity of several characterized Raman bands for SARS-CoV-2 S exhibited a gently downward trend until 60 °C, and decreased from 60 °C to 200 °C. Compared with SARS-CoV S, the SARS-CoV-2 S exhibited less downward trend and started denaturation from 60 °C, indicating higher temperature tolerance of the Raman activity for SARS-CoV-2 S protein.

**Figure 3:**
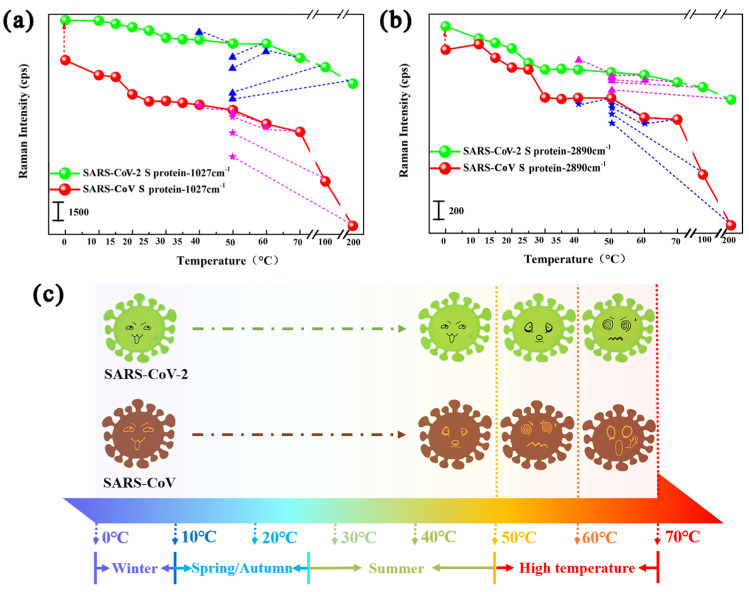
(**a**,**b**) Raman–temperature recovery curves for SARS-CoV-2 S proteins and SARS-CoV S proteins with Raman shifts at 1027 cm^−1^ (**a**) and 2890 cm^−1^ (**b**) which were incubated at gradually increasing temperature (≥50 °C) and then returned to the previous temperature. The triangles and stars represent the Raman peak intensity of SARS-CoV-2 S proteins and SARS-CoV S proteins recovered to an adjacent low-temperature point from a next-stepping higher-temperature point, respectively. (**c**) Schematic diagram of the tolerance of the SARS-CoV-2 and SARS-CoV viruses to temperature changes.

Furthermore, the virus S proteins on the SERS chips were subjected to heating and then cooling treatment to explore the tolerance of the protein structure to temperature changes. The Raman–temperature recovery curve of those proteins that were incubated at gradually increasing temperature (≥50 °C) and then returned to the previous temperature is shown in Figure 3a,b. When the temperature decreased from 50 °C to 40 °C, the Raman intensity of SARS-CoV-2 S protein at the Raman shifts of 1027 cm^−1^ and 2890 cm^−1^ returned to the original strength or above, demonstrating that the protein spatial conformation including secondary and tertiary structure showed no changes. While when the temperature decreased from 50 °C to 40 °C, the Raman intensity of SARS-CoV S protein at the Raman shifts of 1027 cm^−1^ and 2890 cm^−1^ returned below the original Raman intensity, indicating the protein spatial conformation including secondary and tertiary structure began to change. Similarly, when the temperature decreased from 60 °C to 50 °C and 70 °C to 60 °C, the Raman intensity of SARS-CoV-2 S protein at the Raman shifts of 1027 cm^−1^ and 2890 cm^−1^ returned below the original Raman intensity, demonstrating that the spatial conformation of SARS-CoV-2 protein exist denatured under the temperatures of 70 °C and 60 °C. Therefore, the above measured results indicated that SARS-CoV-2 S protein did not denature at around 50 °C and only became denatured at 60 °C, while SARS-CoV S protein began to denature at around 50 °C. In a word, the Raman activity of SARS-CoV-2 S protein exhibited higher temperature tolerance from 0 °C to 60 °C than SARS-CoV S protein, suggesting that the SARS-CoV-2 virus has less temperature influence from increasing air temperature than the SARS-CoV virus to a certain extent, as shown in Figure 3c. The above research conclusion of the variation in the survival ability of the SARS-CoV-2 virus with temperature verifies the fact that the SARS-CoV-2 virus does not disappear with the increase in summer temperature like the SARS-CoV virus, and also indicates that the SARS-CoV virus will accelerate its inactivation in high-temperature environments, which also explains the regional transmission pattern of the SARS-CoV-2 virus such that the high-incidence areas of COVID-19 epidemic are mostly concentrated in temperate regions with an average temperature of 5–11 °C, and the transmission strength is relatively low in high-temperature areas (such as Southeast Asia).

**Figure 4:**
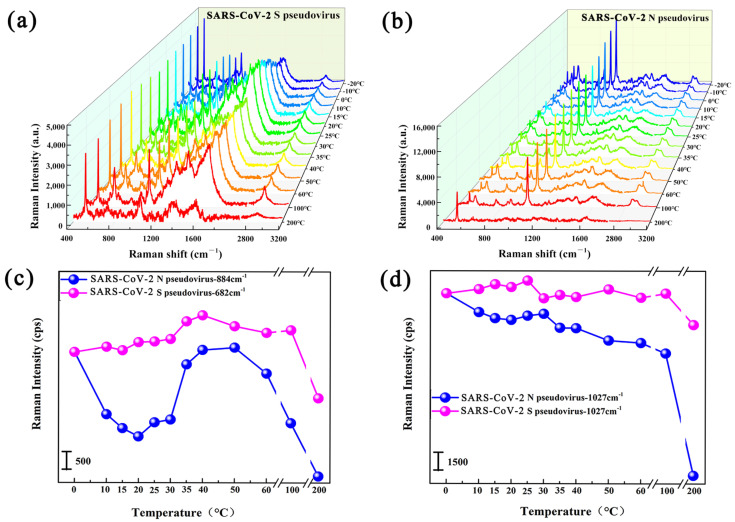
(**a**,**b**) Raman spectra of SARS-CoV-2 S pseudovirus (**a**) and SARS-CoV-2 N pseudovirus (**b**) measured at temperatures ranging from 0 °C to 200 °C. (**c**,**d**) The Raman intensity changes of SARS-CoV-2 S pseudovirus and SARS-CoV-2 N pseudovirus with temperatures ranging from 0 °C to 200 °C at the Raman shifts of (**c**) 884 cm^−1^/682 cm^−1^, (**d**) 1027 cm^−1^.

Furthermore, the SARS-CoV-2 S pseudovirus and N pseudovirus were adopted to verify the tolerance of the SARS-CoV-2 virus to temperature changes, and the detected SERS-enhanced spectra are shown in Figure 4a,b. Analysis of the relationship between Raman intensity and temperature reveals that the Raman intensities of the SARS-CoV-2 S pseudovirus and N pseudovirus do not show a unified variation pattern with temperatures in the temperature range of 0–60 °C, which might be related to the complex viral structures of the SARS-CoV-2 S pseudovirus and N pseudovirus. However, when the temperature rises to 100 °C and 200 °C, the Raman intensity of both the SARS-CoV-2 S pseudovirus and SARS-CoV-2 N pseudovirus shows a sharp downward trend at temperatures from 60 °C to 200 °C, as shown in Figure 4c,d. Moreover, the SARS-CoV-2 S pseudovirus with the surface S protein also exhibits higher temperature tolerance compared to the SARS-CoV-2 N pseudovirus without S protein. The above results on SARS-CoV-2 pseudovirus also indicate that the SARS-CoV-2 virus exhibits higher temperature tolerance than the SARS-CoV virus.

## 4. Conclusions

This work focuses on the assessment of the environmental survival ability of viruses based on SERS technology. Au nanocone arrays fabricated on silicon substrates through a simple Ar ion sputtering route are applied as ultra-sensitive SERS chips for detecting SARS-CoV-2 S proteins at different temperatures, which exhibit a low LOD of 1 pg/mL and a high SERS EF of 4.89 × 10^9^. Raman spectra of SARS-CoV-2 and SARS-CoV S protein under temperatures ranging from 0 °C to 200 °C indicated that SARS-CoV-2 S protein exhibited higher temperature tolerance from 0 °C to 60 °C than SARS-CoV S protein, suggesting that the SARS-CoV-2 virus has less temperature influence from the increasing air temperature than the SARS-CoV virus to a certain extent. Furthermore, after heating and then cooling treatment with SARS-CoV-2 and SARS-CoV S protein on SERS chips, the Raman–temperature recovery curves revealed that SARS-CoV-2 S protein did not denature at around 50 °C and only became denatured at 60 °C, while SARS-CoV S protein began to denature at around 50 °C. The above research explains the seasonal recurrence pattern and regional transmission pattern of the SARS-CoV-2 virus which is different from that of the SARS-CoV virus, and also verifies the feasibility of applying SERS technology to explore viral tolerance to temperature changes, which is of important guiding significance for effective epidemic prevention and control as well as virus inactivation treatment of unknown and sudden highly contagious and highly pathogenic viruses in the future.

## Data Availability

The original contributions presented in this study are included in the article/Supplementary Materials. Further inquiries can be directed to the corresponding author.

## Supplementary Materials

The following supporting information can be downloaded at: https://www.mdpi.com/article/10.3390/bios15090558/s1, Figure S1: Raman spectra of R6G with concentrations of 10^−5^–10^−10^ M on Au nanoarray SERS chips and Raman intensity of R6G on Au nanoarray SERS chips at 1647 cm^−1^ as a function of its concentration of 10^−5^–10^−10^ M; Table S1: Summarizing table of Raman intensity changes across temperatures for both SARS-CoV-2 and SARS-CoV S proteins. References [38,39] are cited in the Supplementary Materials.
