# Supplementary material for: Exploration of the Tolerance of Novel Coronaviruses to Temperature Changes Based on SERS Technology
Source: Biosensors (Basel). 2025 Aug 22;15(9):558. doi: 10.3390/bios15090558 (PMC12467563; doi:10.3390/bios15090558)
Supplement: Supplementary file 1 [file biosensors-15-00558-s001.zip › biosensors-3768498-supplementary.pdf]

biosensors

Exploration on the Tolerance of the Novel Coronavirus to Temperature Changes Based on SERS Technology

Yusi Peng [1,2], Shuai Zhao [1,2,3], Masaki Tanemura [4], Yong Yang [1,2,*] and Ming Liu [5,*]

[1] State Key Laboratory of High-Performance Ceramics and Superfine Microstructures, Shanghai Institute of Ceramics, Chinese Academy of Sciences, 1295 Dingxi Road, Shanghai 200050, China; pengyusi@mail.sic.ac.cn (Y.P.); zhaoshuai211@mails.ucas.ac.cn (S.Z.)
[2] Center of Materials Science and Optoelectronics Engineering, University of Chinese Academy of Sciences, Beijing 100049, China
[3] University of Chinese Academy of Sciences, No.19 (A) Yuquan Road, Beijing 100049, China
[4] Department of Frontier Materials, Graduate School of Engineering, Nagoya Institute of Technology, Showa, Nagoya 466-8555, Japan; tanemura.masaki@nitech.ac.jp
[5] Department of Orthopedics, Shanghai Fourth People's Hospital, School of Medicine, Tongji University, Shanghai 200333, China
* Correspondence: yangyong@mail.sic.ac.cn (Y.Y.); 2105765@tongji.edu.cn (M.L.)

Enhancement factor (EF) calculations

SERS enhancement factors (EFs) of Au nanoarrays SERS chips for SARS-CoV-2 S protein were calculated by the following general formula [1]:

$$\mathrm{EF}=\frac{\mathrm{I_{SERS}}}{\mathrm{I_{prob}}}\times\frac{\mathrm{N_{prob}}}{\mathrm{N_{SERS}}} \qquad \text{(S1)}$$

Based on formula (1), I_{SERS} and I_{prob} are the Raman intensity at a selected Raman peak of SARS-CoV-2 S protein on SERS chips and SARS-CoV-2 S protein. N_{SERS} is the average number of SARS-CoV-2 S protein on SERS chips in the Raman detection region. N_{prob} is the average number of SARS-CoV-2 S protein powder in the Raman detection region. With respect to the average number of SARS-CoV-2 S protein SERS enhanced by Au nanoarrays SERS chips N_{SERS}:

$$\mathrm{N_{SERS}}=\frac{\mathrm{C_{sol}V_{sol}N_AA_{Raman}}}{\mathrm{A_{substrate}}}=\frac{\mathrm{C_{sol}h_{sol}A_{substrate}N_AA_{Raman}}}{\mathrm{A_{substrate}}}=\mathrm{C_{sol}h_{sol}N_AA_{Raman}} \qquad \text{(S2)}$$

As for the average molecular number of SARS-CoV-2 S protein powder without SERS enhancement N_{prob}:

$$N_{prob}=C_{prob}h_{prob}N_AA_{Raman} \qquad \text{(S3)}$$

Where A_{Raman} is the laser radiation area. h_{prob} and h_{sol} are the depth of laser radiation. As for the SARS-CoV-2 S protein powder samples, the laser radiation depth h_{prob} is approximately 21 μm [2]. With respect to the low-concentration SARS-CoV-2 S protein samples, Raman Mapping scanning technology was applied for detection. Since the SARS-CoV-2 S protein on the SERS chips is usually unevenly distributed, not every detection point will show the Raman signals of SARS-CoV-2 S protein. Therefore, the laser radiation depth h_{SERS} of detection points that show the Raman signals of SARS-CoV-2 S protein can be considered to be approximately equal toh_{prob}. C_{sol} (molL^{-1}) is the concentration of the detected SARS-CoV-2 S protein. C_{prob} is the molar concentration of SARS-CoV-2 S protein powder:

 https://doi.org/10.3390/bios15090558

$$C_{prob} = \frac{m/M}{V_{prob}} = \frac{\rho_{prob}}{M} = \frac{1.41g/mL}{M} \quad (S4)$$

$$\frac{N_{prob}}{N_{SERS}} = \frac{C_{prob}h_{prob}N_A A_{Raman}}{C_{sol}h_{sol}N_A A_{Raman}} = \frac{\frac{\rho_{prob}}{M}h_{prob}}{\frac{\rho_{sol}}{M}h_{sol}} = \frac{\rho_{prob}h_{prob}}{\rho_{sol}h_{sol}} \quad (S5)$$

Therefore, the SERS enhancement factor is:

$$\text{EF}=\frac{I_{SERS}}{I_{prob}} \times \frac{N_{prob}}{N_{SERS}} = \frac{I_{SERS}}{I_{prob}} \times \frac{1.41}{\rho_{sol}} \quad (S6)$$

According to the SERS enhanced spectra of SARS-CoV-2 S protein with a concentration of 1 ng/mL, the Raman intensity I_{prob} of SARS-CoV-2 S protein powder at 1248 cm^{-1} is 53903. With respect to the SERS enhancement of 1 ng/mL SARS-CoV-2 S protein on SERS ships, the I_{SERS} is 187024. Therefore, EF=4.89×10^9.

Production and purification of SARS-CoV-2 spike protein-based pseudovirions

Pseudovirions were produced by co-transfection of 293T cells with SARS-CoV-2 S pike rotein expressing vector pcDNA3.1(+)-Opt-S and packaging vector pNL4-3-luc+R-E- through polyetherimide (PEI). The supernatants were harvested at 48 h post-transfection, passed through 0.45 μm filter and centrifuged at 800×g for 5 min to remove cell debris. In order to get purer pseudovirions, 5×PEG8000 NaCl solution was added to the collected pseudovirions, left at 4℃ overnight, and centrifuged at 4000 g for 20 min next day. The supernatant was finally removed and collected by using 40 μl PBS solution.

SERS performance characterization of Au nanoarrays

The R6G aqueous solution with the different concentration of 10^{-5}-10^{-10} M were used to investigate the SERS performance of Au nanoarrays. For each Raman test, a dose of R6G aqueous solution with a volume of 10 μL was dropped on the surface of Au nanoarrays SERS chips and dried at room temperature. All the Raman spectra of dye molecules were obtained by Renishaw inVia Reflex Raman spectrometer with the laser power of 5 mW 532 nm and the accumulation time was 20 s, and the laser beam was focused to a spot about 2 μm in diameter with a 50× microscope objective. At least three different points on each substrate were tested, and selected the medium intensity of the Raman spectra at 1647 cm^{-1} peak to analyze the relationship trend between the Raman intensity and the R6G concentration.

Table S1. Summarizing table of Raman intensity changing across temperatures for both SARS-CoV-2 and SARS-CoV S protein.

Temperature	SARS-CoV-2 S protein			SARS-CoV S protein		
	884 cm^{-1}	1027 cm^{-1}	2890 cm^{-1}	752 cm^{-1}	1027 cm^{-1}	2890 cm^{-1}
**0℃**	1113	6689	1028	1980	16901	2245
**10℃**	635	6636	872	1696	15411	2317
**15℃**	610	6331	816	1404	15239	2143
**20℃**	505	5986	746	1056	13499	2018
**25℃**	375	5647	559	986	12798	1993
**30℃**	410	4942	475	917	12831	1635
**35℃**	338	4782	484	1083	12645	1616
**40℃**	324	4742	473	1017	12448	1629
**50℃**	140	4344	440	916	11924	1627

**60℃**	119	4332	407	668	10536	1378
**70℃**	106	2931	312	374	9741	1354
**100℃**	100	1994	250	330	4788	649
**200℃**	0	347	90	128	342	0

Note: In order to more clearly compare the Raman intensity variation trend of SARS-CoV-2 and SARS-CoV S protein with temperature, the Raman intensity of SARS-CoV-2 S protein was normalized by using the Raman intensity of SARS-CoV S protein at 0℃ as the standard, thereby forming the intensity trend varying with temperature as shown in Figure 2(d-f).

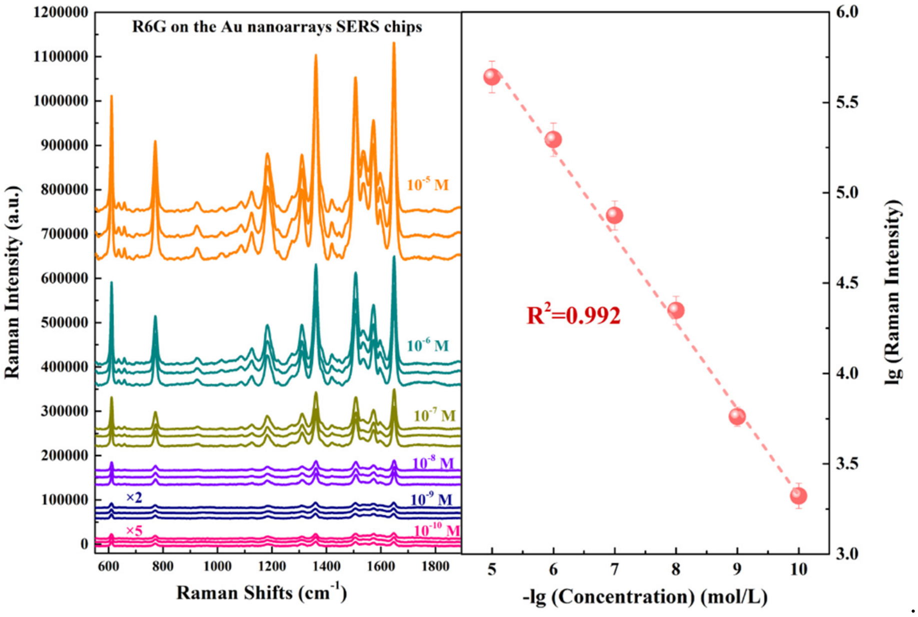

Figure S1. Raman spectra of R6G with concentration of 10^{-5}-10^{-10} M on Au nanoarrays SERS chips. And Raman intensity of R6G on Au nanoarrays SERS chips at 1647 cm^{-1} as a function of its concentration of 10^{-5}-10^{-10} M.

Reference

1. Sarycheva, A.; Makaryan, T.; Maleski, K.; Satheeshkumar, E.; Melikyan, A.; Minassian, H.; Yoshimura, M.; Gogotsi, Y. Two-Dimensional Titanium Carbide (MXene) as Surface-Enhanced Raman Scattering Substrate. *J. Phys. Chem. C* **2017**, 121(36), 19983-19988.
2. Yang, L.; Peng, Y. S.; Yang, Y.; Liu, J. J.; Huang, H. L.; Yu, B. H.; Zhao, J. M.; Lu, Y. L.; Huang, Z. R.; Li, Z. Y.; Lombardi, J. R. A Novel Ultra-Sensitive Semiconductor SERS Substrate Boosted by the Coupled Resonance Effect. Adv. Sci. **2019**, 6(12), 1900310.
